# Associations Between Severe Influenza‐Complicated Thromboembolism Events, Intensive Care Unit Stays and Mortality, and Associated Risk Factors: A Retrospective Cohort Study

**DOI:** 10.1111/irv.13354

**Published:** 2024-08-27

**Authors:** Wei‐Chun Lee, Che‐Chia Chang, Meng‐Chin Ho, Chin‐Kuo Lin, Chieh‐Mo Lin, Yu‐Hung Fang, Shu‐Yi Huang, Yu‐Ching Lin, Min‐Chun Chuang, Tsung‐Ming Yang, Ming‐Szu Hung, Yen‐Li Chou, Ying‐Huang Tsai, Meng‐Jer Hsieh

**Affiliations:** ^1^ Department of Pulmonary and Critical Care Medicine Chiayi Chang‐Gung Memorial Hospital, Chang‐Gung Medical Foundation Chiayi Taiwan; ^2^ Department of Medicine, School of Medicine Chang Gung University Taoyuan Taiwan; ^3^ Department of Respiratory Care Chang Gung University of Science and Technology Chiayi Taiwan; ^4^ Department of Pulmonary and Critical Care Medicine Linkou Chang‐Gung Memorial Hospital, Chang‐Gung Medical Foundation Taoyuan Taiwan; ^5^ Department of Respiratory Therapy, School of Medicine Chang‐Gung University Taoyuan Taiwan

**Keywords:** community‐acquired pneumonia, influenza, intensive care stay, mortality, risk factors, thromboembolism disease

## Abstract

**Abstract:**

The association between influenza infection and thromboembolism (TE) events, including cardiovascular events, cerebrovascular events, pulmonary embolism, and deep vein thrombosis, is supported by compelling evidence. However, there is a disparity in the risk factors that impact the outcomes of severe influenza‐complicated TE in intensive care unit (ICU) patients. The objective of this study was to evaluate the outcomes of severe influenza‐complicated TE in ICU patients and identify any associated risk factors.

**Methods:**

A retrospective cohort study was conducted, recruiting consecutive patients with TE events admitted to the ICU between December 2015 through December 2018 at our institution in Taiwan. The study included a group of 108 patients with severe influenza and a control group of 192 patients with severe community‐acquired pneumonia. Associations between complicated TE, length of ICU stay, and 90‐day mortality were evaluated using logistic regression analysis, and risk factors were identified using univariate and multivariate generalized linear regression analyses.

**Results:**

TE event prevalence was significantly higher in ICU patients with severe influenza than in ICU patients with severe CAP (21.3% vs. 5.7%, respectively; *p* < 0.05). Patients with severe influenza who developed TE experienced a significant increase in the ratio of mechanical ventilation use, length of mechanical ventilation use, ICU stay, and 90‐day mortality when compared to patients without TE (all *p* < 0.05). The comparison of severe CAP patients with and without TE revealed no significant differences (*p* > 0.05). The development of thromboembolic events in patients with severe influenza or severe noninfluenza CAP is linked to influenza infection and hypertension (*p* < 0.05). Furthermore, complicated TE and the severity of the APACHE II score are risk factors for 90‐day mortality in ICU patients with severe influenza (*p* < 0.05).

**Conclusions:**

Patients with severe influenza and complicated TE are more likely to have an extended ICU stay and 90‐day mortality than patients with severe CAP. The risk is significantly higher for patients with a higher APACHE II score. The results of this study may aid in defining better strategies for early recognition and prevention of severe influenza‐complicated TE.

**Abbreviations**
APACHE: acute physiology and chronic health evaluation
BMI: body mass index
CAP: community‐acquired pneumonia
CKD: chronic kidney disease
COPD: chronic obstructive pulmonary disease
CVD: cardiovascular disease
EORTC/MSG: Research and Treatment of Cancer/Mycoses Study Group
ESRD: end‐stage renal disease
TB: tuberculosis
TE: thromboembolism

## 1 Introduction

Arterial thromboembolism (TE) (acute myocardial infarction (AMI) or ischemic stroke) and venous TE (deep vein thrombosis (DVT) or pulmonary embolism), known as cardiovascular disease (CVD), are the leading causes of mortality in the world, representing 32% of all global deaths in 2019 [1]. Older adults are most likely to die from CVD, which accounts for 38% of all deaths in that age group. Epidemiologic data reveal that CVD is a major health burden among older adults in terms of functional decline, overall mortality, and economic impact [2].

The high global burden of CVD has made identifying and mitigating modifying risk factors a priority for prevention. Hypertension is the primary risk factor for myocardial infarction development, followed by tobacco use, and high serum cholesterol ranks sixth [3]. The role of infection in triggering vascular events has also been investigated, and influenza is the most reliable evidence [4]. The pathogenesis of thrombosis typically occurs as a result of endovascular injury or endothelial activation caused by the elaboration of a variety of proinflammatory mediators with subsequent platelet and coagulation pathway activation during influenza infection. Influenza is a major cause of hospitalization and death for infected patients, with over 20,000 annual influenza deaths worldwide [5]. Compelling evidence points to the association between influenza infection and AMI, and both influenza and cardiac deaths peak in winter [6, 7]. In addition, influenza epidemics are associated with increased hospitalization rates for AMI and other cardiovascular‐related conditions [8]. A number of retrospective and prospective studies have shown a strong association between influenza and AMI [4, 8, 9, 10, 11], showing a temporal relationship, with influenza respiratory illnesses preceding AMI by varying times. Recently, a large survey of patients with AMI found that postrespiratory infection accounts for 10% of all patients and is associated with poorer prognosis and twice the rate of in‐hospital mortality [11].

However, there has been no study that has shown an association between influenza and the incidence and outcomes of other acute TE events, including ischemic stroke, DVT, and pulmonary embolism, in patients with severe influenza infection in the intensive care unit (ICU). The risk factors that affect the outcomes of severe influenza complicating TE in these patients are unknown. We aimed to assess the incidence of major vascular complications in patients with severe influenza infection under intensive care and to determine whether severe influenza infection‐related vascular complications influenced the risk of longer ICU stays, longer mechanical ventilation use, and mortality. We hypothesized that identifying corresponding risk factors may help to define better strategies for prevention and early recognition of severe influenza‐complicated TE and help to identify patients requiring intensive care to mitigate the potential negative outcomes. This study was designed to evaluate the outcomes of severe influenza‐complicated TE in ICU patients and to identify the risk factors involved.

## 2 Patients and Methods

### 2.1 Study Design and Population

The study is a retrospective cohort study of ICU‐hospitalized patients treated from December 2015 through December 2018 at our institution in Chiayi, Taiwan. The study protocol was approved by the institutional review board of the Chang Gung Memorial Hospital, Taiwan. The patients were categorized into a study cohort for severe influenza and a control group of patients with severe community‐acquired pneumonia (CAP) according to their diagnosis. Individuals diagnosed with severe influenza were admitted to the ICU from outside the hospital after presenting with respiratory insufficiency or unstable vital signs due to influenza infection. A positive airway PCR test (PCR QiAamp Viral RNA Mini Kit, TAIGEN Bioscience Corporation, Taiwan) was used to define influenza as a confirmed influenza infection. Inclusion criteria were patients older than 18 years who were admitted to the ICU for more than 24 h with acute respiratory failure and had pulmonary infiltrates on imaging. Patients with healthcare‐associated pneumonia or hospital‐acquired pneumonia, as defined in the American Thoracic Society/Infectious Diseases Society of America (ATS/IDSA) guidelines [12], those for whom respiratory failure was not the primary indication for ICU admission, and those with a confirmed alternative diagnosis lasting until the end of the follow‐up were excluded.

### 2.2 Primary and Secondary Outcomes

Outcomes were selected based on representative influenza outcomes used for clinical assessment in interventional studies of influenza [13, 14]. The primary outcomes were complicated CVD, the length of ICU and hospital stays, and mortality rates (28 and 90 days). Secondary outcomes included the duration of mechanical ventilator support, and any composites consisting of all or some of these outcomes. Ventilator support was defined as the need for respiratory support beyond applying oxygen alone. Data of body mass index (BMI), age, gender, history of TB or other microbe infections, steroid use, comorbidities (diabetes, liver cirrhosis, CKD, end‐stage renal disease (ESRD) under hemodialysis, chronic obstructive pulmonary disease (COPD), asthma, cancer malignancy, prior CVD, hypertension, dyslipidemia, and autoimmune disease), and admission Acute Physiology and Chronic Health Evaluation (APACHE) II scores [15] were also collected.

### 2.3 Measures of TE Incidence

Incidence data were obtained from patients' medical records on the date of complicated TE diagnosis and the International Statistical Classification of Diseases, Tenth Revision (ICD‐10) codes for the underlying cause of death. Daily incidence of TE (DVT, pulmonary embolism, acute coronary syndrome (ACS), and stroke) or daily mortality counts for all‐cause deaths in our hospital were collected.

### 2.4 Statistical Analysis

Results are presented as frequency and percentage or median, unless otherwise indicated. The standard distribution of continuous variables was tested using Student's *t* test, while the Mann–Whitney *U* test was used for nonparametric analyses. The Chi‐square test and Fisher's exact test were used for categorical variables. Risk factors for the incidence and mortality in ICU patients with severe influenza were evaluated using univariate and multivariate logistic regression analyses. Univariate and multivariate generalized linear regression analyses were used to evaluate risk factors for the length of ICU stay in these patients. Variables showing significance in univariate analysis (*p* < 0.05) were included in the multivariate logistic regression analysis using the backward elimination method. The 95% confidence intervals (CIs) for all comparisons are also reported. A two‐tailed *p*‐value of < 0.05 was considered to indicate statistical significance. The Statistical Package for the Social Sciences statistical software (version 26.0; SPSS Inc, Chicago, IL, USA) was used for all statistical analyses.

## 3 Results

### 3.1 Patients' Characteristics

**FIGURE 1:**
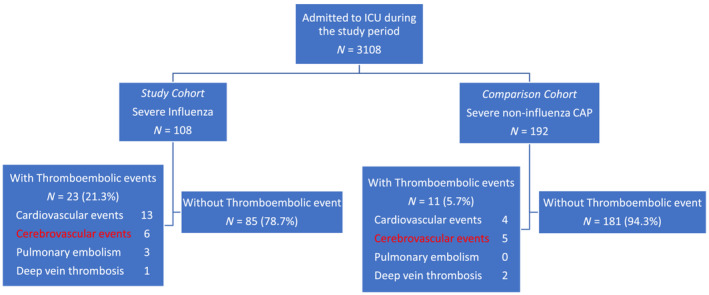
Distribution of patients with severe influenza pneumonitis and severe noninfluenza CAP.

The data on a total of 300 ICU patients were included in the study, with 108 ICU patients with severe influenza as the study cohort and 192 ICU patients with severe CAP as the comparison cohort (Figure 1). The baseline characteristics of the two groups are summarized in Table 1. The influenza and CAP groups had a median age of 68.5 years and 71.9 years, respectively, with a *p* = 0.078. Cardiovascular risk factors were present in both groups, with 38% of individuals having diabetes, 13% having dyslipidemia, and 57% having hypertension in the influenza group, while 48% had diabetes, 16% had dyslipidemia, and 31% had hypertension in the CAP group. Comparing with CAP groups, ICU patients with severe influenza had lower APACHE II score (mean 17.4 and 20.4, respectively, *p* < 0.05), lower mechanical ventilation support (68.5% and 97.9%, respectively, *p* < 0.001), lower rate progress to ARDS (20% and 56%, respectively, *p* < 0.001), low acute kidney injury (14.8% and 56.3%, respectively, *p* < 0.001), higher incidence of TE (21.3% and 5.73%, respectively, *p* < 0.05), longer ICU stay (mean 20.85 and 19.45, respectively, *p* < 0.05), and lower 90‐day mortality (22.2% and 32.4%, respectively, *p* < 0.05). Besides, Table 2 summarizes the baseline characteristics of patients with or without TE events between CAP and influenza groups. Among ICU patients with severe influenza, 23 (21%) cases with complicated TE had a median age of 70 years, and males were the largest proportion (57%). Among those without complicated TE, 85 (79%) had median ages of 71 years, and males were the largest proportion (62%). Compared with non‐TE patients, patients with severe influenza had more comorbidities of hypertension (83% and 52%, respectively, *p* < 0.05) and dyslipidemia (26% and 9%, respectively, *p* < 0.05). Among ICU patients with severe CAP, 11 (5.7%) cases with complicated TE had a median age of 71 years, and a larger proportion was male (82%). Among 181 cases (94.3%) without complicated TE, the median age was 72 years and a larger proportion was male (73%). Hypertension was significantly higher in CAP patients with TE (82% and 46% respectively, *p* < 0.05).

**TABLE 1:** Demographic data of patients with severe influenza infection or severe noninfluenza community‐acquired pneumonia in the ICUs.

	Severe influenza *n* = 108	Severe noninfluenza CAP *n* = 192	*p*‐value
Gender (M/F)	66/42	142/50	0.021*
Age (year, mean ± SD)	68.49 ± 16.25	71.88 ± 14.05	0.078
BH (cm, mean ± SD)	160 (143–184, 15)	161 (135–182, 12)	0.117
BW (kg, mean ± SD)	61.5 (30–109, 18)	56 (20–97, 17)	0.007*
BMI (mean ± SD)	23.25 (13.5–40.2, 5.15)	21.75 (13.5–34.5, 5.5)	0.001*
APACHE II score (mean ± SD)	17.38 ± 6.52	20.35 ± 6.02	< 0.001*
Time to TE events (days) (mean ± SD)	12.00 ± 14.03	8.45 ± 5.36	0.565
Use of MV, *N* (%)	74 (68.5%)	188 (97.9%)	< 0.0001*
MV days (mean ± SD)	17.22 ± 16.06	20.06 ± 18.97	0.404
ICU days (mean ± SD)	20.85 ± 19.46	19.45 ± 13.93	0.001*
Hospital days (mean ± SD)	22.34 ± 19.41	35.44 ± 28.92	< 0.001*
Use of ECMO, *N* (%)	5 (1.6%)	1 (0.5%)	0.052
ARDS	22 (20%)	108 (56%)	< 0.001*
Acute kidney injury	16 (14.8%)	102 (53%)	< 0.001*
Invasive aspergillosis infection	10 (9.3%)	21 (10.9%)	0.648
**Thromboembolic events**, *N* (%)	23 (21.3%)	11 (5.73%)	< 0.001*
Cardiovascular	13	4	
Cerebrovascular	6	5	
Pulmonary embolism	3	0	
Deep vein thrombosis	1	2	
28‐day mortality, *N* (%)	20 (18.52%)	46 (23.96%)	0.275
90‐day mortality, *N* (%)	24 (22.22%)	68 (35.42%)	0.017*
***Comorbidities***, *N* (%)			
Cerebrovascular	7 (6.48%)	25 (13.02%)	0.078
Cardiovascular	19 (17.59%)	45 (23.44%)	0.236
Pulmonary embolism history	0 (0%)	1 (0.52%)	1.000
Hypertension	63 (58.33%)	93 (48.44%)	0.100
Dyslipidemia	14 (12.96%)	31 (16.15%)	0.459
COPD	22 (20.37%)	21 (10.94%)	0.025*
Asthma	6 (5.56%)	6 (3.13%)	0.302
ICS	11 (10.19%)	6 (3.13%)	0.011*
OCS	2 (1.85%)	6 (3.13%)	0.777
TB	1 (0.93%)	11 (5.73%)	0.118
LC	10 (9.26%)	16 (8.33%)	0.784
Diabetes mellitus	41 (37.96%)	60 (31.25%)	0.238
Chronic kidney disease	8 (7.41%)	15 (7.81%)	0.899
ESRD under HD	6 (5.56%)	3 (1.56%)	0.111
Active cancer	9 (8.33%)	27 (14.06%)	0.143
Chemotherapy	3 (2.78%)	6 (3.13%)	1.000
Biologics	0 (0%)	3 (1.56%)	0.483
Autoimmune disease	1 (0.93%)	9 (4.69%)	0.159
AIDS	0 (0%)	1 (0.52%)	1.000
Malnutrition	0 (0%)	2 (1.04%)	0.287

Abbreviations: AIDS: acquired immunodeficiency syndrome; APACHE II: Acute Physiology and Chronic Health Evaluation II score; ARDS: acute respiratory distress syndrome; BH: body height; BMI: body mass index; BW: body weight; CAP: community‐acquired pneumonia; COPD: chronic obstructive pulmonary disease; ECMO: extracorporeal membrane oxygenation; ESRD: end‐stage renal disease; HD: hemodialysis; ICS: inhaled corticosteroid; LC: liver cirrhosis; MV: mechanical ventilation; OCS: oral corticosteroid; TB: tuberculosis; TE: thromboembolic.

* *p* < 0.05, Chi‐square test, Mann–Whitney *U* test.

**TABLE 2:** Baseline characteristics in patients with or without thromboembolism events.

	Severe influenza*n* = 108			Severe noninfluenza CAP*n* = 192		
	With TE events *n* = 23	Without TE events *n* = 85	*p*‐value	With TE events *n* = 11	Without TE events *n* = 181	*p*‐value
**Age**, mean ± SD	70.26 ± 15.59	68.01 ± 16.48	0.602	71 ± 10.23	71.93 ± 14.27	0.548
**Gender**, *n* (%)			0.611			0.541
Male	13 (56.52%)	53 (62.35%)		9 (81.82%)	133 (73.48%)	
Female	10 (43.48%)	32 (37.65%)		2 (18.18%)	48 (26.52%)	
**BMI (kg/m** ^**2**^ **)**	24.14 ± 4.52	23.82 ± 4.91	0.907	24.61 ± 4.28	21.81 ± 4.32	0.056
**APACHE II score**, mean ± SD	17.74 ± 6.43	17.28 ± 6.58	0.710	20.82 ± 4.64	20.32 ± 6.11	0.658
**Oral corticosteroid**, *n* (%)	0 (0)	2 (2.36%)	1.000	0 (0)	6 (3.31%)	1.000
**Comorbidities**, *n* (%)						
Hypertension	19 (82.61%)	44 (51.76%)	0.008*	9 (81.82%)	84 (46.41%)	0.029*
Dyslipidemia	6 (26.09%)	8 (9.41%)	0.035*	2 (18.18%)	29 (16.02%)	0.693
Prior vascular events	8 (34.78%)	16 (18.82%)	0.102	5 (45.45%)	62 (34.25%)	0.449
Diabetes	10 (43.48%)	31 (36.47%)	0.539	5 (45.45%)	55 (30.39%)	0.295
Liver cirrhosis	1 (4.35%)	9 (10.59%)	0.686	1 (9.09%)	15 (8.29%)	1.000
COPD	4 (17.39%)	18 (21.18%)	0.779	1 (9.09%)	20 (11.05%)	1.000
Asthma	1 (4.35%)	5 (5.88%)	1.000	0 (0)	6 (3.31%)	1.000
Prior TB infection	1 (4.35%)	0 (0)	0.213	1 (9.09%)	10 (5.50%)	0.646
CKD	2 (8.7%)	6 (7.06%)	0.678	0 (0)	15 (8.29%)	1.000
ESRD under hemodialysis	2 (8.7%)	4 (4.71%)	0.606	0 (0)	3 (1.66%)	1.000
Cancer	2 (8.7%)	7 (8.24%)	1.000	0 (0)	27 (14.92%)	0.368
Autoimmune disease	0 (0)	1 (1.18%)	1.000	0 (0)	9 (4.97%)	1.000

Abbreviations: APACHE, acute physiology and chronic health evaluation; BMI, body mass index; CAP, community‐acquired pneumonia; CKD, chronic kidney disease; COPD, chronic obstructive pulmonary disease; CVD, cardiovascular disease; ESRD, end‐stage renal disease; TB, tuberculosis.

* *p* < 0.05, *Chi‐square test*, Mann–Whitney *U* test.

### 3.2 Risk Factors for Developing TE

Table 1, Table 2, and Figure 1 show that ICU patients with severe influenza had a greater likelihood of developing TE compared to patients with severe CAP (21.3% and 5.7%, respectively; *p* < 0.05). Subsequent multivariate logistic regression analysis showed that influenza infection (OR: 4.018, 95% CI = 1.799–8.974, *p* < 0.05) and hypertension (OR: 3.634, 95% CI = 1.065–12.402, *p* < 0.05) were significant independent risk factors contributing to the incidence of TE in ICU patients with severe influenza (Table 3). Prior hypertension (OR: 6.157, 95% CI = 1.161–32.637, *p* < 0.05) and higher BMI (OR: 1.158, 95% CI = 1.007–1.332, *p* < 0.05) significantly increased the risk of incident TE in ICU patients with severe CAP (Table 3).

**TABLE 3:** Multivariate logistic regression of the risk factors for thromboembolic events in patients with severe influenza or severe noninfluenza CAP.

Factors	Risk for thromboembolic events		
	OR	95% CI	*p*‐value
**Multivariate analysis**			
*All patients*			
Influenza vs. noninfluenza CAP	4.018	1.799–8.974	0.001*
Gender (female vs. male)	0.809	0.349–1.872	0.620
BMI	1.044	0.964–1.131	0.291
Hypertension	4.475	1.667–12.031	0.003*
Dyslipidemia	1.094	0.397–3.012	0.862
Diabetes mellitus	1.084	0.491–2.293	0.841
*Severe influenza pneumonitis patients*			
Gender (female vs. male)	1.328	0.460–3.834	0.599
BMI	0.980	0.888–1.082	0.700
Hypertension	3.634	1.065–12.402	0.039*
Dyslipidemia	2.770	0.694–11.057	0.149
Diabetes mellitus	0.882	0.318–2.446	0.809
*Severe noninfluenza CAP patients*			
Gender (female vs. male)	0.329	0.062–1.747	0.192
BMI	1.158	1.007–1.332	0.039*
Hypertension	6.157	1.161–32.637	0.032*
Dyslipidemia	0.457	0.083–2.503	0.367
Diabetes mellitus	1.052	0.273–4.047	0.940

Abbreviations: 95% CI: 95% confidence interval; APACHE II: Acute Physiology and Chronic Health Evaluation II score; BMI: body mass index; CAP: community‐acquired pneumonia; OR: odds ratio; TE: thromboembolic.

* *p* < 0.05.

### 3.3 Cumulative Incidence of Developing TE

**FIGURE 2:**
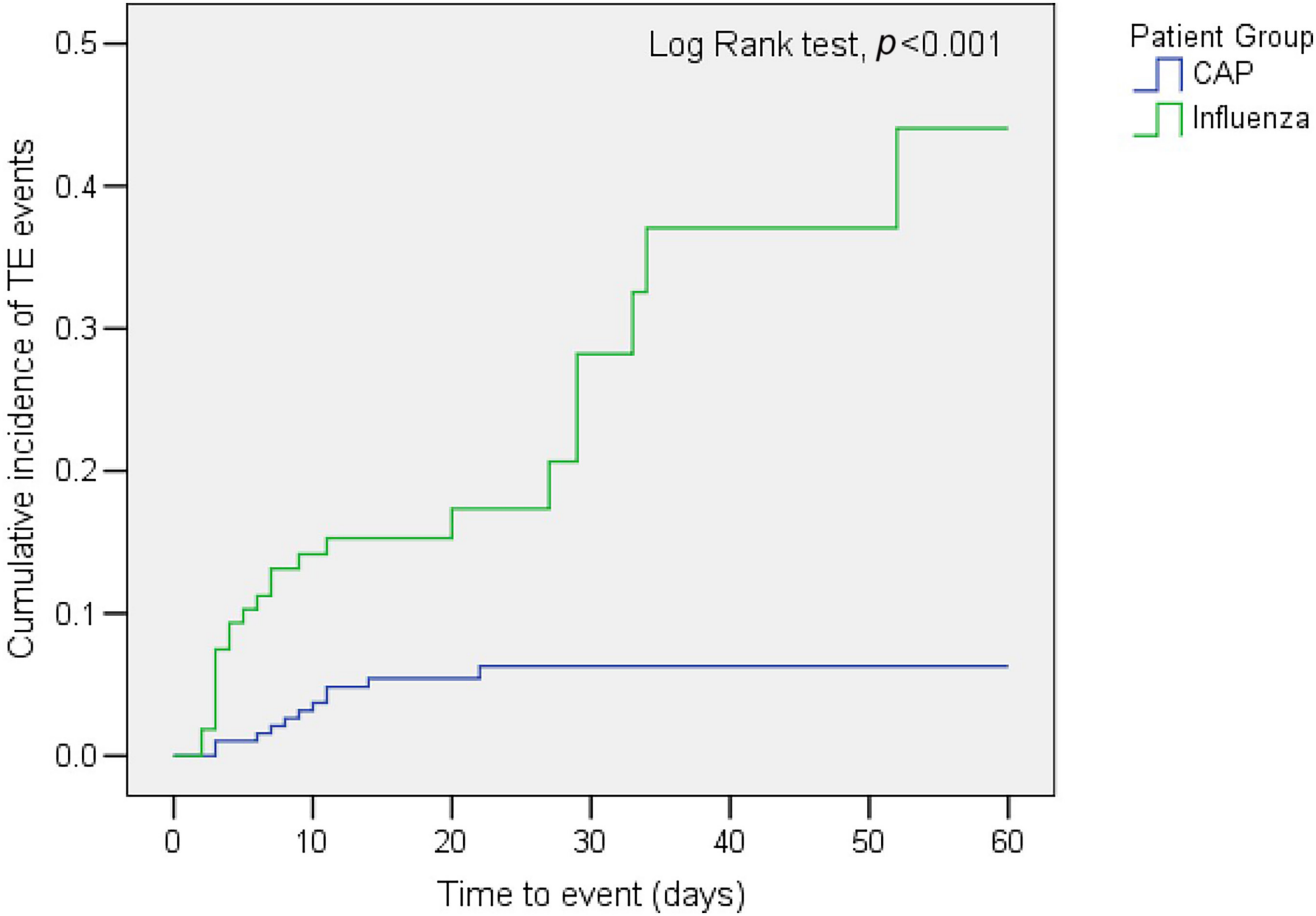
Time to the occurrence of thromboembolic events in the patients with severe influenza or severe noninfluenza CAP admitted to the ICUs.

Figure 2 shows the time to the occurrence of thromboembolic events in the patients with severe influenza or severe noninfluenza CAP. The cumulative incidence of developing TE was significantly higher in the severe influenza group (*p* < 0.001).

### 3.4 Relationships Between TE and ICU Outcomes

The use of mechanical ventilation, duration of mechanical ventilation use, ICU stays, ICU mortality, and 90‐day mortality increased significantly from baseline in ICU patients with severe influenza after developing TE compared with those parameters in patients who did not develop TE (all *p* < 0.05, Table 4). No significant differences were noted in hospital stay and 28‐day mortality between the two groups (all *p* > 0.05, Table 3). In contrast, the duration of mechanical ventilation use, ICU stay, hospital stay, 28‐day mortality, and 90‐day mortality was not significantly different between CAP patients with and without developmental TE (all *p* > 0.05, Table 4).

**TABLE 4:** Impacts of acute TE events on patients' outcomes.

	Severe influenza			Severe noninfluenza CAP		
	With TE events *n* = 23	Without TE events *n* = 85	*p*‐value	With TE events *n* = 11	Without TE events *n* = 181	*p*‐value
**Use of mechanical ventilation**	23 (100%)	51 (60%)	< 0.001*	11 (100%)	177 (97.80)	0.618
**Length of mechanical ventilation (days)**	20.52 ± 13.98	15.44 ± 16.78	0.009*	20.36 ± 14.11	20.41 ± 19.26	0.420
**ICU stay (days)**	34.47 ± 28.53	14.44 ± 8.54	< 0.001*	18.18 ± 7.83	19.53 ± 14.23	0.644
**Hospital stay (days)**	26.87 ± 26.03	21.64 ± 16.78	0.707	33.27 ± 20.61	35.57 ± 29.38	0.971
**ICU mortality**	5 (21.7%)	8 (9.4%)	0.07864	3 (23.1%)	30 (16.8%)	0.228
**28‐day mortality**	7 (30.43%)	13 (15.29%)	0.097	3 (27.27%)	43 (23.76%)	0.727
**90‐day mortality**	11 (47.83%)	13 (15.29%)	0.001*	3 (27.27%)	65 (35.91%)	0.749

Abbreviations: CAP, community‐acquired pneumonia; CVD, cardiovascular disease; ICU, intensive care unit.

* *p* < 0.05, *Chi‐square* test, Mann–Whitney *U* test.

### 3.5 Risk Factors for 28‐Day Mortality and 90‐Day Mortality

Table 5 shows the risk factors associated with 28‐day and 90‐day mortality. The risk of 28‐day and 90‐day mortality in all ICU patients was increased by developing to ARDS and TE events, as shown by multivariate logistic regression analysis (all *p* < 0.05). In addition, patients with a higher APACHE II score had a higher risk of dying within 90 days (OR: 1.077, 95% CI 1.023–1.135, *p* < 0.05). In the influenza group, higher APACHE II score and invasive aspergillosis infection were independent risk factors for 28‐day and 90‐day mortality (all *p* < 0.05). Besides, age, TE event, and acute kidney injury were all risk factors for 90‐day mortality in the influenza group (all *p* < 0.05). On the contrary, diabetes was a protective factor for both 28‐day and 90‐day mortality in influenza patients (all *p* < 0.05). During CAP groups, ARDS was the independent factor in both 28‐day and 90‐day mortality (OR: 12.694, 95% CI: 3.484–46.252, and OR: 6.222, 95% CI 2.663–14.535, respectively, both *p* < 0.001). Additionally, a higher APACHE II score is associated with higher 90‐day mortality rates in this group (OR: 1.084, 95% CI 1.014–1.159, *p* < 0.05).

**TABLE 5:** Multivariate logistic regression of the risk factors for 28‐day and 90‐day mortality in patients with severe influenza or severe noninfluenza CAP.

	28‐day mortality			90‐day mortality		
	OR	95% CI	*p*‐value	OR	95% CI	*p*‐value
**Multivariate analysis**						
*All patients*						
Influenza vs. noninfluenza CAP	0.678	0.282–1.629	0.385	0.926	0.420–2.037	0.848
Age	0.999	0.975–1.024	0.944	1.020	0.996–1.045	0.107
APACHE II	1.053	0.977–1.112	0.064	1.077	1.023–1.135	0.005*
ARDS	3.520	1.643–7.546	0.001*	3.908	1.975–7.732	< 0.001*
TE events	2.702	1.003–7.283	0.049*	3.581	1.423‐9.012	0.007*
Invasive aspergillosis infection	1.321	0.495–3.519	0.578	0.902	0.339–2.404	0.837
Diabetes mellitus	0.696	0.330–1.469	0.342	0.530	0.268–1.050	0.069
Acute kidney injury	1.916	0.917–4.002	0.084	1.644	0.848–3.264	0.138
*Severe influenza pneumonitis patients*						
Age	1.054	0.995–1.116	0.073	1.076	1.011–1.146	0.022*
APACHE II	1.138	1.005–1.288	0.042*	1.127	1.006–1.264	0.040*
ARDS	0.503	0.054‐4.669	0.545	1.205	0.187–7.766	0.844
TE events	4.470	0.776–25.750	0.094	27.044	3.393–215.523	0.002*
Invasive aspergillosis infection	27.864	2.377–326.166	0.008*	27.6.2	2.27–280.044	0.005*
Diabetes mellitus	0.042	0.004–0.405	0.006*	0.075	0.011‐0.504	0.008*
Acute kidney injury	5.249	0.770–35.798	0.091	6.736	1.085–41.830	0.041*
*Severe noninfluenza CAP patients*						
Age	0.977	0.946–1.009	0.152	1.006	0.978–1.035	0.672
APACHE II	1.052	0.977–1.131	0.177	1.084	1.014–1.159	0.018*
ARDS	12.694	3.484–46.252	< 0.001*	6.222	2.663–14.535	< 0.001*
TE events	2.060	0.389–10.905	0.395	1.238	0.277–5.534	0.780
Invasive aspergillosis infection	1.415	0.364–5.497	0.616	2.316	0.671–7.999	0.184
Diabetes mellitus	1.403	0.525–3.750	0.500	0.905	0.390–2.101	0.817
Acute kidney injury	2.104	0.842–5.256	0.111	1.357	0.622–2.960	0.444

Abbreviations: 95% CI: 95% confidence interval; APACHE II: Acute Physiology and Chronic Health Evaluation II score; BMI: body mass index; CAP: community‐acquired pneumonia; OR: odds ratio; TE: thromboembolism.

* *p* < 0.05.

## 4 Discussion

Our study showed that influenza‐complicated TE in ICU patients is associated with an increased risk of longer duration of mechanical ventilation, extended ICU stays, and 90‐day mortality. A recent study found that hospitalized patients with influenza infection had a 90‐day presence of an absolute risk of arterial TE and venous TE, with 14.4% and 5.3%, respectively [16]. Furthermore, a comparable investigation revealed the prevalence of venous TE in patients who have SARS‐CoV‐2 infection, influenza, and CAP [17]. Our study, unlike the previous studies, concentrated on the occurrence of TE, the risk factors associated with it, and the outcomes of complicated TE in patients with severe influenza and CAP who were treated in ICUs.

A retrospective evaluation of the data of 300 ICU patients with severe influenza and CAP showed that ICU patients with severe influenza infection had a higher incidence of major vascular complications than did patients with CAP (Table 1). Besides, the cumulative incidence of developing TE was significantly higher in severe influenza groups than in CAP groups (Figure 2). Significant increases in mechanical ventilation use, ICU stays, ICU mortality, and 90‐day mortality were observed in influenza patients with incident major vascular complications. These increases were not observed in CAP patients (Table 4). Among ICU patients with severe influenza, the presence of prior hypertension significantly increased the risk of developing TE, suggesting that hypertension is an independent risk factor influencing the development of TE. Our study also presented that developing to TE, invasive aspergillosis infection, and acute kidney injury was among independent risk factors for 90‐day mortality in patients with influenza, while diabetes mellitus was an independent protective factor in 90‐day mortality (Table 5).

Previous studies have also reported associations between CVD and viral infections. A study of pandemic 2009 influenza A (H1N1) reported that only 4.9% of all patients hospitalized for H1N1 had left ventricular dysfunction, but among patients in that study who underwent echocardiography, 25% had evidence of left ventricular dysfunction [18]. Evidence of myocarditis was found in 21.7% of patients who are sicker, in the ARDS death group, in a Chinese study of severe cases of H1N1 2009 [19], who were diagnosed using clinical, laboratory, and echocardiographic criteria. Evaluation of myocardial injury and dysfunction in 37 patients admitted to the ICU with severe H1N1 infection revealed myocardial injury in 17 patients (46%), and left ventricular systolic dysfunction in 20 patients (54%) [20]. In that study, diagnosis of myocarditis was presumed in 14 (37.8%) patients who had myocardial injury and global myocardial dysfunction in the setting of an acute viral infection. The present study expanded previous findings and further showed that ICU patients with severe influenza (87% influenza A virus) had a high frequency (21.3%) of developing TE events, including DVT (1.0%), pulmonary embolism (2.8%), ACS (7.4%), stress cardiomyopathy (5.6%), and stroke (4.6%). As such, the present study is the first to demonstrate associations between severe influenza and pulmonary embolism, stress cardiomyopathy, and DVT in ICU patients with severe influenza, although complicated DVT was observed in H1N1 infection in a hospitalized adolescent patient [21]. Also, the association between influenza infection and AMI has been well described in previous research [6]. Cardiac involvement in influenza is reported to occur between 4 and 9 days after the onset of influenza symptoms [22, 23], which is compatible with our result of a median of 5 days from the onset of identification of cardiac events. It has been observed that the majority of patients experienced complicated TE within 5 days after mechanical ventilation was initiated. It is important to consider the possibility of pre‐existing heart disease and cardiac complications when managing influenza infection, especially because of the increased mortality associated with these diagnoses [20, 24].

Furthermore, arterial hypertension is a recognized cardiovascular risk factor for the onset of peripheral vascular disease [25], cerebrovascular disease [26], and coronary artery disease [27]. As previously mentioned, prior hypertension played an independent role in the development of TE in ICU patients with severe influenza and CAP group in the present study. The challenge of treating severe influenza means that patients with comorbid hypertension may have an even higher incidence of TE and a higher risk of ICU admission or mortality.

Previous evidence has revealed that high cardiovascular mortality occurs mostly in winter and is associated with the influenza peak, which strongly suggests a relationship between influenza infection and ACS [9]. The risk of myocardial infarction and stroke was significantly higher in a large cohort of older adults during the first 3 days following the diagnosis of respiratory infections [28]. More recently, influenza infection has been linked to a higher risk of cardiovascular mortality within 14 days and strongly correlates with fatal ACS [29]. Similarly, the present study demonstrated that ICU patients with severe influenza infection‐complicated TE had a higher 90‐day mortality rate than those without complicated TE (Table 5). According to multivariate logistic regression analysis in this study, complicated TE is a predictive factor for 90‐day mortality in ICU patients with severe influenza. It is suggested that TE incidence may be linked to influenza infection, leading to an increase in 90‐day mortality rates among ICU patients with severe influenza. Furthermore, our study demonstrated that infection with invasive aspergillosis and the development of acute kidney injury were independent risk factors for mortality in ICU patients with severe influenza (Table 5). Invasive pulmonary aspergillosis (IPA) was diagnosed in 19% of ICU patients with influenza infection, complicated by an almost double 90‐day mortality rate than patients without IPA (51% vs. 28%, adjusted odds ratio [OR] 1.87, 95% CI 1.05–3.32) [30]. A previous study also found that 33.6% of ICU patients with severe influenza infection had acute kidney injury, which was independently associated with an increased risk of hospital mortality. Our data showed that IPA was diagnosed in 9.3% of the influenza group and that there was an increased risk of both 28‐day and 90‐day mortality.

It is noteworthy that our study suggested that diabetes mellitus is a factor that protects against both 28‐day and 90‐day mortality in individuals with influenza. Although DM has been known as one risk factor associated with poor outcomes in critically ill patients, a lower mortality rate in patients with DM was noted among a population of patients experiencing sepsis that was reported in one prospective study [31]. In another retrospective study, the mortality rate was significantly lower among patients with herpes simplex virus pneumonitis in critically ill patients with diabetes mellitus (OR: 0.12, 95% CI: 0.02–0.49, *p* = 0.0009) [32]. The reason why DM is presented as a protective factor remains unclear. One possible reason is that patients with diabetes can tolerate hyperglycemia to a greater extent than patients without diabetes. Therefore, patients with diabetes experience less harm from the blood sugar level fluctuations that can occur during critical illness [33]. Another possible explanation is that in Taiwan, the recommendation for influenza vaccination is given to patients with diabetes mellitus. A previous meta‐analysis study demonstrated the effectiveness of influenza vaccination in reducing hospitalization and mortality in patients with DM [34]. Further investigation is necessary to confirm this mechanism.

Complicated TE and increased cardiovascular mortality are also noted in other virus‐caused respiratory diseases, such as SARS, MERS, and the novel coronavirus disease 2019 (COVID‐19) [35]. Several studies have indicated that COVID‐19 contributes to acute cardiac injury, shock, arrhythmia, stress cardiomyopathy, ventricular tachycardia/fibrillation, and myocarditis [35, 36]. COVID‐19 patients who were admitted to ICU experienced a significant increase in the prevalence of cardiometabolic disease compared to non‐ICU patients. A recent study presented no significant difference in the risk of arterial TE among those hospitalized with COVID‐19 during either period (adjusted hazard ratios, 1.04 and 1.07) vs. those hospitalized with influenza [16]. Furthermore, the incidence of acute cardiac injury was approximately 13 times higher in ICU cases than in non‐ICU cases [37]. Compared with COVID‐19, the influences of CVD and hypertension on the case fatality rate are lower in influenza [35]. Nevertheless, the association of cardiovascular comorbidities with the mortality is still highly positive [35]. As a result, specific cardiovascular considerations, including antiarrythmic management, anticoagulation, and hemodynamic support, are important and necessary for treating severe virus‐caused respiratory diseases, especially for patients who are admitted to the ICU. We also compared patients who had an initial COVID‐19 infection admitted to our ICU between May 1, 2022, and August 31, 2022. A total of 202 patients were analyzed during vaccine availability. The result indicated no significant increase in the incidence of atrial TE events and mortality among COVID‐19 and influenza groups (15.8% and 17.6%, respectively, *p* = 0.455, 53.1% and 43.4%, respectively, *p* = 0.489).

Influenza is thought to act through many mechanisms, including inflammatory release of cytokines that causes a prothrombotic state, local disruption of coronary plaque, as well as physiological effects such as hypoxia and tachycardia, ultimately causing acute obstruction of the coronary arteries [38]. Other mechanisms may include sympathetic activation, which may result in vasoconstriction, thrombogenesis through nonspecific procoagulant and thrombophilic effects of inflammation, epithelial dysfunction, reduced coronary artery blood flow through increased metabolic demand with fever and tachycardia, and reduced oxygen saturation and hypotension with secondary vasoconstriction [39]. Influenza has also been shown to have direct effects on the heart. An influenza‐infected mice model showed that the virus can be isolated from heart tissue and that its presence leads to local inflammatory changes [40]. Besides, the contributions of inflammation by exploring the nuanced cellular interactions between immune responses and coagulation, including hemostasis, immunothrombosis, deranged thromboinflammation, vessel barrier dysfunction, leukocyte contributions, neutrophil extracellular traps, platelet adhesion, and coagulation pathways, result in VTE [41]. Based on these findings and the results of the present study, we suggest that further investigations are needed to determine whether influenza‐related DVT, pulmonary embolism, ACS, stress cardiomyopathy, and stroke arise from these mechanisms. Furthermore, a recent meta‐analysis has revealed that receiving influenza vaccination is associated with a 34% lower risk of major adverse cardiovascular events, and individuals with recent ACS have a 45% lower risk [42]. Therefore, clinicians should continue counseling their high‐risk patients on the cardiovascular benefits of seasonal influenza vaccination.

### 4.1 Limitations

The present study has several limitations, including that the analysis of data was retrospective, which limits the inference of causality, and it was also a single‐center study, which limits the possibility of generalization to other populations. Secondly, this study was conducted mainly in respiratory ICUs, which requires that patient characteristics be interpreted in that context. Also, due to the relatively small cohort, the statistical power of the observation of median onset of TE events in ICU patients with severe influenza is limited, although our results compared favorably to the results of other studies.

### 4.2 Conclusions

Influenza‐complicated TE in ICU patients with severe influenza infection is associated with outcomes such as increased risk of longer duration of mechanical ventilation, extended ICU stays, and 90‐day mortality. Incidents of severe influenza‐related TE may include DVT, pulmonary embolism, ACS, stress cardiomyopathy, and stroke. Complicated TE and TE‐related ICU stays are more likely to occur in this patient population when hypertension is present. The results of this study could lead to better strategies for early recognition and prevention of severe influenza‐complicated TE, particularly in individuals with comorbid hypertension.

## Author Contributions

**Wei‐Chun Lee:** conceptualization, methodology, writing–original draft. **Che‐Chia Chang:** data curation, investigation. **Meng‐Chin Ho:** conceptualization, methodology. **Chin‐Kuo Lin:** data curation. **Chieh‐Mo Lin:** data curation, investigation. **Yu‐Hung Fang:** data curation. **Shu‐Yi Huang:** data curation, investigation. **Yu‐Ching Lin:** data curation, investigation. **Min‐Chun Chuang:** data curation. **Tsung‐Ming Yang:** data curation, investigation. **Ming‐Szu Hung:** data curation, investigation. **Yen‐Li Chou:** data curation, investigation. **Ying‐Huang Tsai:** conceptualization, methodology. **Meng‐Jer Hsieh:** conceptualization, methodology, supervision, writing–review and editing.

## Ethics Statement

This study was approved by the Institutional Review Board of Chang‐Gung Medical Foundation (IRB No.: 202001202B0).

## Consent

Informed consent was waived by the IRB.

## Conflicts of Interest

The authors declare no conflicts of interest.

### Peer Review

The peer review history for this article is available at https://www.webofscience.com/api/gateway/wos/peer‐review/10.1111/irv.13354.

## Data Availability

The authors confirm that the data supporting the findings of this study are available within the article.
